# The Role of Nanocurcumin in Nanoplastics-Induced Pathological Alterations in Largemouth Bass (*Micropterus salmoides*)

**DOI:** 10.3390/antiox15070829

**Published:** 2026-06-30

**Authors:** Tengfei Zhu, Yongxin Liu, Mingshi Chen, Yamin Wang, Wenjie Chu, Shuling Bai, Qi Li, Zhipeng Zheng, Hao Chen, Jiandong Zhu, Yingying Yu, Dianchang Zhang

**Affiliations:** 1 Key Laboratory of South China Sea Fishery Resources Exploitation and Utilization, Ministry of Agriculture and Rural Affairs, South China Sea Fisheries Research Institute, Chinese Academy of Fishery Sciences, Guangzhou 510300, China; 2 Sanya Tropical Fisheries Research Institute, Sanya 572018, China; 3 Guangdong Provincial Key Laboratory of Animal Molecular Design and Precise Breeding, School of Animal Science and Technology, Foshan University, Foshan 528225, China; 4 Guangdong Provincial Engineer Technology Research Center of Marine Biological Seed Industry, Guangzhou 510300, China

**Keywords:** nanocurcumin, histopathological alterations, oxidative stress, lipid metabolism, intestinal microbiota

## Abstract

Nanoplastics (NPs) are emerging aquatic pollutants that may threaten farmed fish health. This study evaluated whether dietary nanocurcumin (NCUR) modulates NP-associated biological disturbances in largemouth bass (*Micropterus salmoides*). A total of 480 fish (initial weight 11.52 ± 0.02 g) were assigned to four treatments for 21 days: control, 0.2% NCUR, 100 μg/L NPs, and 0.2% NCUR + 100 μg/L NPs. NP exposure significantly reduced growth and feed intake (*p* < 0.05) and was accompanied by gill, hepatic and intestinal tissue alterations, disturbed serum lipid parameters, changes in hepatic antioxidant enzyme activities, and altered mRNA expression of antioxidant- and metabolism-related genes. Dietary NCUR was associated with partial modulation of several NP-associated responses, including hepatic and intestinal structural alterations, serum lipid changes, adaptive antioxidant enzyme responses, and transcriptional changes in Nrf2/Keap1- and SIRT1/FoxO1-associated genes. However, NCUR did not alleviate NPs-induced gill damage, and NCUR alone also caused branchial histopathological alterations. Therefore, 0.2% NCUR may partially mitigate selected NPs-induced disturbances in *M. salmoides*, but its independent effects, branchial safety, and pathway-level mechanisms require further investigation.

## 1. Introduction

Plastic products are used widely with the advantages of low cost, light weight, and great plasticity and chemical stability. However, the mishandling of plastic products has resulted in numerous plastic waste discharges into the natural environment. These plastics undergo degradation through complex physical, chemical, and biological transformation processes, forming microplastics (MPs, with diameters less than 5 mm), which further fragment into nanoplastics (NPs, with diameters less than 100 nm) [1,2]. Owing to the stable chemical properties and resistance to degradation of NPs, they exhibit long-term environmental persistence in the natural environment and are widely distributed across marine, terrestrial, and atmospheric environmental media through surface runoff, wind transport and precipitation, leading to severe pollution of the environment [3,4]. Due to their small particle size, nanoplastics are readily absorbed by aquatic organisms and accumulate in their tissues, thereby exerting adverse effects on organismal fitness. Feng et al. reported that zebrafish (*Danio rerio*) embryos exposed to 100 nm PS-NPs showed decreased hatching and survival rates, inhibited heart rate and body length, and suppressed behavioral activity, indicating that PS-NPs can induce developmental toxicity during early life stages [5]. Another study reported that the addition of 10, 100, and 1000 μg/L PS-NPs (with a particle size of 70 nm) to the aquaculture water for 7 days not only exerted an adverse impact on the locomotor behavior of *Carassius auratus*, but also penetrated the fish epidermis and entered the muscle tissue, impairing its nerve fibers and suppressing the activity of AChE [6]. Du et al. reported that exposure to 1000 μg/L PS-NPs disturbed hepatic lipid metabolism and intestinal microbiota stability in juvenile zebrafish (*Danio rerio*), and the combined exposure of PS-NPs and a high-fat diet further aggravated gastrointestinal injury [7]. Exposure to 2.5 μg/mL PS-NPs (with a particle size of 50 nm) in the aquatic environment for 14 days could induce oxidative stress in juveniles of *Oryzias melastigma* [8]. A study found that PS-NPs (with a particle size of 70 nm) exposed for 30 days could accumulate in the gonad, intestinal tract, brain and liver of zebrafish, and the supplementation of 5 ppm PS-NPs in the aquatic water for 7 weeks could induce the dysregulation of circadian locomotor rhythm [9]. Zhou et al. reported that exposure to 100 nm PS-NPs affected intestinal digestive enzyme activities, caused slight intestinal structural damage, and altered intestinal microbiota composition in the Asian swamp eel (*Monopterus albus*), suggesting that PS-NPs can impair intestinal health in fish [10].

Largemouth bass (*Micropterus salmoides*) is an economically important farmed freshwater carnivorous fish in China, whose farming industry holds a prominent position in the aquacultural economy. A previous study has confirmed the presence of nanoplastic residues in largemouth bass. Though its growth performance did not alter significantly when the fish were exposed to water containing polystyrene nanoplastics (PS-NPs, with a particle size of 100 nm) with a concentration of 10–100 μg/L, distinct histopathological alterations were identified such as gill lamella fusion, hepatocyte vacuolization, and intestinal villus damage, concomitant with liver antioxidant system dysfunction including abnormal SOD and CAT activity and malondialdehyde (MDA) accumulation and intestinal microbiota structural reconstruction [11]. These pathological lesions directly threaten healthy aquaculture and the quality and safety of largemouth bass products. At present, research on nanoplastic toxicity mitigation technologies for freshwater carnivorous fish remains limited, and effective pollution prevention and control measures for nanoplastics in aquaculture environments are lacking. To further promote the sustainable and healthy development of aquaculture and ensure the quality and safety of aquatic products, it is particularly important to explore a method that can alleviate the damage caused by nanoplastics to fish.

The potential mitigation strategies of feed additives are expected to alleviate NPs-induced toxic effects on aquatic organisms. As a natural plant extract, curcumin exhibits excellent antioxidant [12], hypolipidemic [13], hypoglycemic [14] and anti-inflammatory effects [15]. The phenolic hydroxyl groups and methoxy groups in the molecular structure of curcumin are the key factors for its antioxidant capacity, among which phenolic hydroxyl groups possess the function of capturing or scavenging free radicals, and phenolic hydroxyl groups enhance the capacity of free radical capture and scavenging [16]. Furthermore, curcumin exerts a regulatory effect on primary antioxidant enzymes (such as SOD, GSH-px and CAT) in organisms [17]. A study on *LitoPenaeus vannamei* reported that supplementation of conjugated linoleic acid or curcumin in a diet containing 200 μg/kg aflatoxin reduced the activities of alkaline phosphatase (ALP) in the hepatopancreas, thereby protecting *L. vannamei* from aflatoxin-induced damage [18]. Adding 15 g/kg curcumin to the diet could effectively inhibit the accumulation of Pb in the tissues of *Cyprinus carpio*, alleviating waterborne lead exposure-induced oxidant stress, enhancing immunity, intestinal enzyme activity and improving intestinal microbiota [19]. Another study found that the addition of 100 mg/kg curcumin could alleviate the increase in the content of MDA in *C. carpio* induced by chlorpyrifos. Zhao et al. [20] reported that adding 100 mg/kg curcumin to the diet could alleviate chlorpyrifos-induced oxidant damage in *M. salmoides* to a certain extent; therefore, the ameliorative effect of curcumin was attenuated as the duration of chlorpyrifos exposure increased. Lei et al. [21] found that curcumin (concentration more than 500 μmol/L) could mitigate PS-MPs-induced oxidant stress in *Caenorhabditis elegans* by inhibiting the downregulation of the *gst4* gene. However, its practical application is limited due to its poor water solubility and low bioavailability. It is effective to enhance the solubility, bioavailability and bioactivity of curcumin in water by encapsulating it with liposomes [22].

However, currently, research on NCUR’s influence on aquaculture as a feed additive remains scarce, and its potential benefits and independent biological effects in aquatic fish merit investigation. Therefore, in this trial, we fed *M. salmoides* with a 0.2% NCUR-supplemented diet and exposed them to aquatic water containing 100 μg/L PS-NPs. Growth performance, serum biochemical parameters, hepatic antioxidant-related gene expression, histopathological characteristics and intestinal microbiota were then determined after a 21-day trial to preliminarily evaluate the effects of NCUR as a feed additive on NP-associated biological disturbances in *M. salmoides*.

## 2. Materials and Methods

### 2.1. Ethical Statement

The study was approved by the Animal Care and Use Committee of the South China Sea Fisheries Research Institute, Chinese Academy of Fishery Sciences (No. SCSFRI96-253).

### 2.2. Experimental Diets

As presented in Table 1, the specific feed formula is detailed. Based on the results of previous studies conducted by our research group [23], the dietary NCUR supplementation levels in the present experiment were set at 0% (basal diet) and 0.2% (NCUR-supplemented diet), corresponding to 0 and 2 g of NCUR per kilogram of feed, respectively. All feed ingredients were ultra-finely ground, sieved, accurately weighed, thoroughly mixed, and pelleted. After drying, the experimental diets were sealed and stored at −20 °C in the dark until used.

### 2.3. Experimental Design and Feeding Management

Juvenile *M. salmoides* were purchased from Foshan Sanshui Baijin Aquatic Seedling Co., Ltd. (Foshan, China). The fish were acclimated for two weeks in an indoor recirculating aquaculture system at the Aquaculture Base of Foshan University using aerated tap water. During the acclimation period, fish were fed a commercial diet (Product No. Q/HZYS 02) purchased from Huzhou Yisheng Feed Co., Ltd. (Huzhou, China) twice daily at 08:00 and 17:00, with continuous aeration provided. Water temperature (24 ± 1 °C), pH (7.5–8.5), dissolved oxygen (>7.0 mg/L), and NH4-N (<0.3 mg/L) were maintained.

The same water source and water-exchange regime were used for all treatment groups throughout the trial. No PS-NPs were intentionally added to the control and NCUR-only groups, whereas the NPs and NCUR + NPs groups were experimentally exposed to 100 μg/L PS-NPs.

The fluorescent polystyrene nanoplastics (PS-NPs; monodisperse green fluorescent microspheres; nominal diameter: 0.1 μm; density: 1.05 g/cm^3^; stock concentration: 10 mg/mL, 1% *w*/*v*; excitation/emission wavelengths: 488/518 nm; coefficient of variation: 3–5%) were purchased from Da’e (Tianjin) Technology Co., Ltd. (Tianjin, China). The particles were supplied as 100 mg fluorescent microspheres dispersed in 10 mL deionized water and stored at 4–8 °C before use. Before exposure, the stock suspension was thoroughly mixed and diluted with aquaculture water to a final concentration of 100 μg/L.

The exposure concentration of 100 μg/L PS-NPs was selected based on our previous study, in which this concentration induced oxidative stress, histopathological alterations, and intestinal microbiota dysbiosis in *M. salmoides* [11]. This concentration is also comparable to environmentally relevant PS-NP concentrations used in recent aquatic ecotoxicological studies, in which 10 and 100 μg/L 80 nm PS-NPs were applied as environmentally relevant exposure levels [24], although actual nanoplastic concentrations in aquaculture systems may vary depending on water source, culture conditions, and plastic inputs.

Prior to the start of the experiment, fish were fasted for 24 h. A total of 480 healthy juvenile *M. salmoides* with similar body weight (initial mean weight: 11.52 ± 0.02 g) were randomly distributed into twelve 140 L culture tanks. The experiment consisted of four treatment groups: control group (no NPs in water and fed the basal diet), 0.2% NCUR group (no NPs in water and fed the NCUR-supplemented diet), 100 μg/L NPs group (exposed to NPs in water and fed the basal diet), and 0.2% NCUR + 100 μg/L NPs group (exposed to NPs in water and fed the NCUR-supplemented diet). Each treatment had three tank replicates, with 40 fish per tank, and the experiment lasted for 21 days.

Fish were fed to apparent satiation twice daily at 08:00 and 17:00 with the corresponding diet, and feed intake (FI) was recorded for each tank. Dead fish were removed promptly, and the number and weight of mortalities were recorded. Two-thirds of the water in each tank was replaced daily.

### 2.4. Sample Collection

Fish were weighed on days 7, 14, and 21 of the experiment. At the end of the trial, after 24 h of fasting, fish were randomly selected from each tank for sampling according to different analytical endpoints. The fish surface was rinsed with deionized water to remove any adhering particles, and the fish were then anesthetized with 10 mg/L buffered tricaine methanesulfonate (MS-222) and weighed. Two fish were selected from each tank, and their body weight, body length, visceral weight, and liver weight were accurately recorded for the calculation of weight gain rate (WGR), feed conversion ratio (FCR), condition factor (CF), visceral somatic index (VSI), and hepatosomatic index (HSI). Caudal vein blood was collected from 10 fish per tank. The blood samples were allowed to stand at 4 °C and then centrifuged at 3500 rpm for 15 min to separate the serum. The serum was stored at −80 °C for subsequent analysis of serum biochemical parameters. Three fish were randomly selected from each tank for hepatic antioxidant analysis. Liver samples were dissected, immediately frozen in liquid nitrogen, and stored at −80 °C for the analysis of hepatic oxidative stress-related indices. Another three fish were selected from each tank for qPCR analysis, and liver samples were collected, snap-frozen in liquid nitrogen, and stored at −80 °C until RNA extraction. In addition, two fish-level intestinal samples were collected from each tank for intestinal microbiota analysis, immediately frozen in liquid nitrogen, and stored at −80 °C until DNA extraction. Fish sampled from the same tank were considered biological subsamples rather than independent tank-level replicates. One fish was selected from each tank, and the gill, liver, and intestinal tissues were dissected, thoroughly rinsed with phosphate-buffered saline (PBS), and fixed in 10% neutral buffered formalin at room temperature for 24 h for histopathological examination. The remaining one fish in each tank was dissected to isolate liver tissue, which was immediately placed in electron fixation solution, fixed at room temperature for 2 h, and then transferred to 4 °C for subsequent observation of liver ultrastructure. For variables measured from multiple fish within the same tank, the mean value of each tank was calculated and used as the experimental replicate for statistical analysis.

The WGR, SGR, HSI, VSI, CF, FCR and SR were calculated as follows:

Weight gain rate (WGR, %) = 100 × [(FBW − IBW)/IBW],(1)

where IBW (g) and FBW (g) are initial body weight and final body weight (at day seven, fourteen and twenty-one), respectively;

Specific growth rate (SGR, % day^−1^) = 100 × (Ln (FBW) − Ln (IBW))/days; (2)

Hepatosomatic index (HSI, %) = 100 × liver weight/final body weight;(3)

Viscerosomatic index (VSI, %) = 100 × visceral weight/final body weight;(4)

Condition factor (CF, g/cm^3^) = 100 × (final body weight/final body length^3^); (5)

Feed conversion ratio (FCR) = feed consumed/weight gain;(6)

Survival rate (SR) (%) = 100 × (fish number after exposure/fish number before exposure).(7)

### 2.5. Histopathological Analysis

The gill, liver, and intestinal tissue samples fixed in 10% neutral buffered formalin were dehydrated through a graded ethanol series, embedded in paraffin (KD-BM, Jinhua Kedi Instrumental Equipment Co., Ltd., Jinhua, China), and sectioned at 5 μm using a rotary microtome (YD-202, Jinhua Yidi Medical Apparatus Co., Ltd., Jinhua, China). The sections were then stained with hematoxylin and eosin (H&E) and observed under a Nikon DS-Ri2 microscope camera (Nikon, Tokyo, Japan). For ultrastructural observation, liver samples fixed in electron fixation solution were further dehydrated, embedded in resin, and sectioned. The sections were stained with uranyl acetate for 30 min and lead citrate for 15 min, and then examined using a transmission electron microscope.

### 2.6. Serum Biochemical Analysis

The serum total cholesterol (TCHO), triglyceride (TG), high-density lipoprotein cholesterol (HDL-C), low-density lipoprotein cholesterol (LDL-C) contents, as well as the activities of aspartate aminotransferase (AST) and alanine aminotransferase (ALT) were determined using biochemical assay kits from Jiancheng Biotech Co., Ltd. (Nanjing, China)—the corresponding catalog numbers for these kits are A111-1-1 (for TCHO), A110-1-1 (for TG), A112-1-1 (for HDL-C), A113-1-1 (for LDL-C), C010-2-1 (for AST) and C009-2-1 (for ALT), and all determinations were performed in accordance with the manufacturer’s protocols provided with the kits.

### 2.7. Liver Antioxidant Capacity Analysis

Liver tissue was rinsed with pre-cooled 0.9% physiological saline, blotted dry with filter paper, and approximately 0.1 g of liver was weighed; physiological saline was added at a ratio of 1:9 (*w*/*v*) for homogenization, followed by centrifugation at 3000 rpm for 10 min at 4 °C, and the supernatant was collected. The activities of superoxide dismutase (SOD) and catalase (CAT), as well as the contents of reduced glutathione (GSH) and malondialdehyde (MDA) in the supernatant were determined using biochemical assay kits from Jiancheng Biotech Co., Ltd. (Nanjing, China)—the corresponding catalog numbers for these kits are A001-3 (for SOD), A007-1-1 (for CAT), A006-2-1 (for GSH) and A003-1 (for MDA), and all determinations were performed in accordance with the manufacturer’s protocols provided with the kits.

### 2.8. Intestinal Microbiome Analysis

The intestinal tissues were sent to Guangzhou Genedenovo Biotechnology Co., Ltd. (Guangzhou, China) for DNA extraction and 16S rRNA high-throughput sequencing. Using the genomic DNA of intestinal microbiota as the template, PCR amplification was performed with the specific primers 341F (5′-CCTACGGGNGGCWGCAG-3′) and 806R (5′-GGACTACHVGGGTATCTAAT-3′) targeting the V3-V4 region of the 16S rRNA gene. After extraction and purification, the amplified products were quantified using a fluorescence quantitative kit; a sequencing library was constructed based on the quantitative results, and sequencing was completed on the Illumina PE250 sequencing platform. The raw sequencing data were subjected to preprocessing steps such as quality filtering and decontamination before subsequent bioinformatics analysis.

### 2.9. RNA Isolation and qPCR Analysis

The liver RNA of *Micropterus salmoides* was extracted following the instructions of the TransZol Up Plus RNA Kit (Cat. No. ER501-01, TransGen Biotech Co., Ltd., Beijing, China). Subsequently, the integrity of the extracted RNA was detected by 1% agarose gel electrophoresis, and its concentration was determined. RNA reverse transcription was performed under the guidance of the gDNA Removal and cDNA Synthesis SuperMix (Cat. No. AE311-02, TransGen Biotech Co., Ltd., Beijing, China). Finally, the PerfectStart Green qPCR SuperMix (Cat. No. AQ601-02, TransGen Biotech Co., Ltd., Beijing, China) was used for quantitative real-time PCR (qRT-PCR) analysis of the expression levels of specific genes. The amplification program for qRT-PCR was as follows: 94 °C for 30 s, followed by 45 cycles of 94 °C for 5 s and 60 °C for 30 s, and a final melting curve stage of 95 °C for 15 s, 60 °C for 1 min, and 95 °C for 1 s. The primer sequences are listed in Table 2. The β-actin gene was used as the reference gene, and the relative expression levels of target genes were calculated using the 2^−ΔΔCT^ method [23].

### 2.10. Statistical Analysis

The tank was considered the experimental unit for growth performance, feed intake, survival, serum biochemical parameters, hepatic antioxidant indices, and qPCR data. For variables measured from multiple fish within the same tank, individual fish were treated as subsamples, and tank means were used for statistical analysis. Data are presented as mean ± SEM. Normality and homogeneity of variance were checked before analysis. Data were analyzed using two-way ANOVA to evaluate the main effects of NP exposure, NCUR supplementation, and their interaction. When significant differences were detected, Tukey’s HSD test was used for multiple comparisons.

For intestinal microbiota analysis, two fish-level intestinal samples were collected from each tank, and each treatment contained three replicate tanks. These fish-level microbiota samples were used to display within-treatment microbial variation. Fish sampled from the same tank were considered biological subsamples rather than independent tank-level replicates; therefore, microbiota-related results were interpreted cautiously with consideration of the tank-based experimental design. PICRUSt2 (2.6.2) was used to predict microbial functional profiles. Alpha diversity indices and predicted functional profiles were compared among groups, while beta diversity was visualized by PCoA based on weighted UniFrac distances. PCoA-related group patterns were assessed using the Kruskal–Wallis test.

## 3. Results

### 3.1. Growth Performance

As shown in Table 3, no significant differences were observed in IBW among all groups, indicating that the initial fish size was uniform among treatments.

After the 7-day trial, the FBW, WGR, and SGR of the NCUR + NPs group were significantly lower than those of the NCUR group (*p* < 0.05), whereas no significant differences were observed among the control, NPs, and NCUR + NPs groups. HSI, VSI, CF, and FI per fish did not differ significantly among groups at this time point. Consistently, two-way ANOVA showed a significant main effect of NP exposure on FBW, WGR, and SGR after 7 days, while the main effect of NCUR and the NPs × NCUR interaction were not significant (Table 4).

After the 14-day trial, no significant differences were observed in FBW, WGR, SGR, HSI, VSI, CF, or FI per fish among all groups (*p* > 0.05; Table 3). Two-way ANOVA also showed no significant main effects of NCUR or NP exposure and no significant NPs × NCUR interaction for these parameters (Table 4).

After the 21-day trial, the NPs group showed significantly lower FBW, WGR, and SGR than the control and NCUR groups (*p* < 0.05), while the NCUR + NPs group showed intermediate values and did not differ significantly from either the NPs group or the control and NCUR groups (Table 3). FI per fish was significantly lower in the NPs group than in the control group (*p* < 0.05), whereas the NCUR and NCUR + NPs groups showed intermediate values. No significant differences were observed in HSI, VSI, CF, FCR, or SR among groups after 21 days (*p* > 0.05). Two-way ANOVA further showed that NP exposure had significant main effects on FBW, WGR, SGR, and FI per fish after 21 days, whereas the main effect of NCUR and the NPs × NCUR interaction were not significant for these parameters (Table 4).

### 3.2. Histological Examination

After 21 days of waterborne NP exposure and dietary NCUR supplementation, obvious histopathological alterations were observed in multiple tissues of *M. salmoides*.

**Figure 1:**
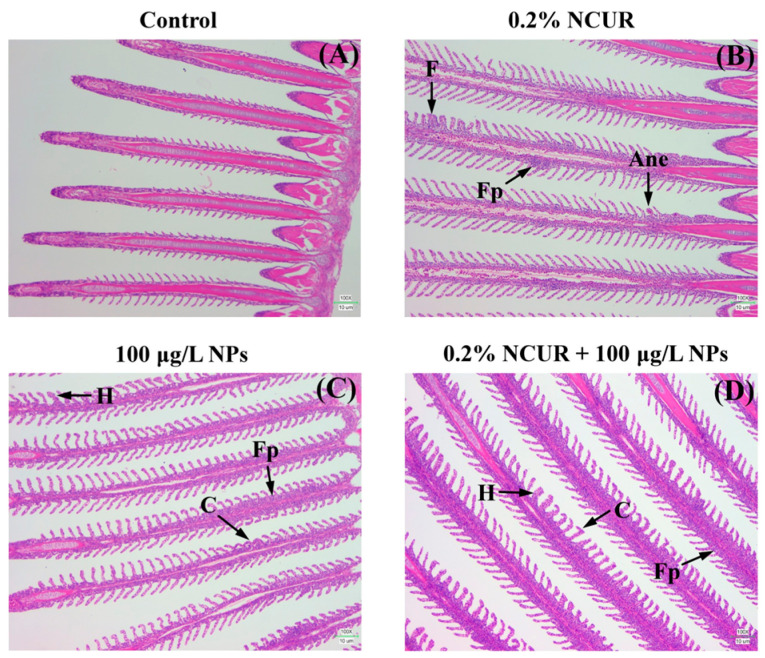
Histopathological changes in gills observed in *M. salmoides* among different treatment groups (Control (**A**), NCUR (**B**), NPs (**C**), NCUR+NPs (**D**)) after 21 days. Hyperplasia (H), curvature (C), aneurysm (Ane), filament epithelium proliferation (Fp), fusion of lamellae (F). Bar = 10 µm, magnification × 100.

In the gills, the control group exhibited a normal histological structure with intact lamellae and no pathological lesions (Figure 1A). In contrast, the NPs group presented obvious gill damage, characterized by gill lamellar curvature, epithelial cell hyperplasia and basal cell hyperplasia (Figure 1C). No apparent alleviation of these pathological changes was observed in the NCUR + NPs group; the gill tissues of this group displayed similar lesions to the NPs group, including lamellar curvature, epithelial cell hyperplasia and basal cell hyperplasia (Figure 1D). Additionally, the NCUR group also showed histopathological alterations in the gills, such as lamellar fusion, basal cell hyperplasia and aneurysm (Figure 1B).

**Figure 2:**
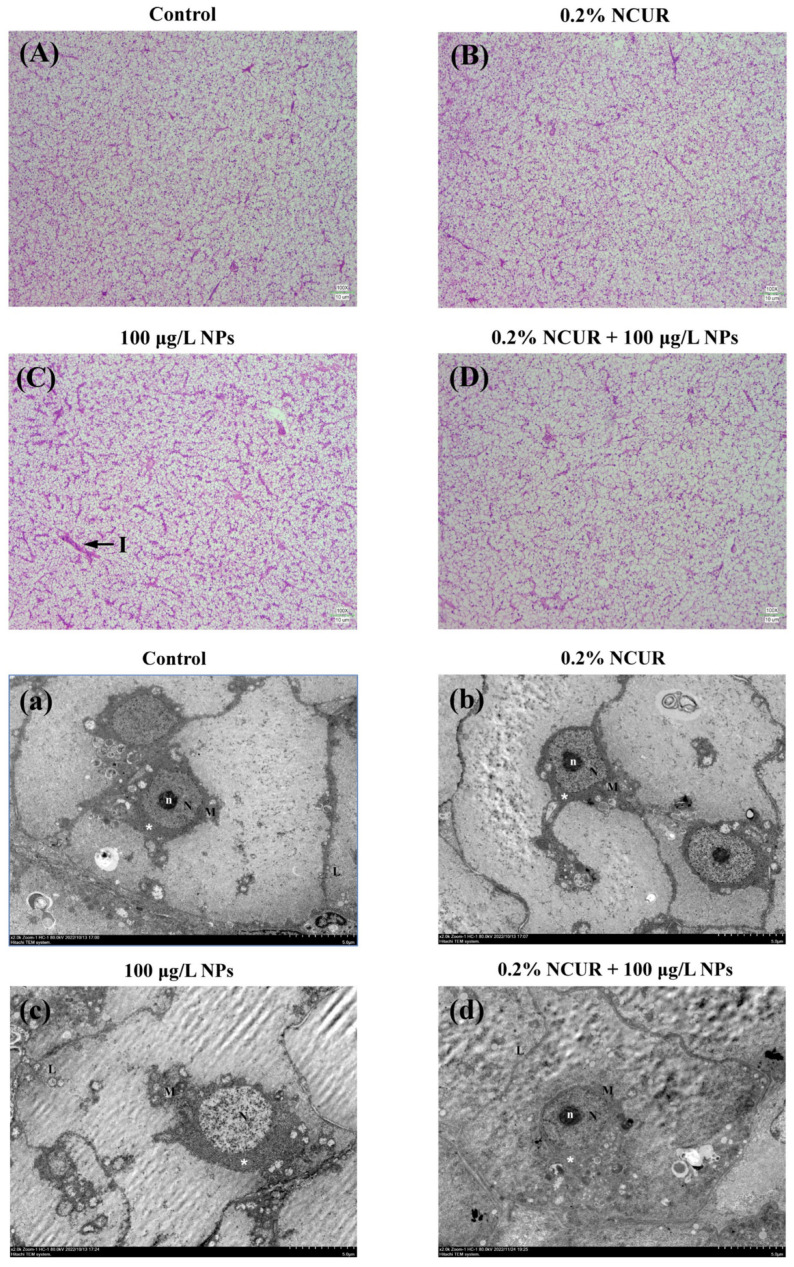
Effects of NP exposure and dietary NCUR supplementation on the liver structure for 21 days. Infiltration of hemocytes (I), Mitochondria (M), rough endoplasmic reticulum (*), nucleus (N), nucleolus (n), lipid droplet (L). (**A**–**D**): Bar = 10 µm, magnification × 100. (**a**–**d**): Bar = 5 µm, magnification × 2000.

As for the liver, the hepatocytes in the control group showed clear boundaries, intact structures, and tight arrangement (Figure 2A), with round-shaped nuclei and evenly distributed chromatin. In addition, a few lipid droplets were detected in the hepatic cytoplasm (Figure 2a). The NCUR group displayed normal hepatic structure, with clear boundaries, intact hepatocytes (Figure 2B), and round-shaped nuclei, similar to the control group (Figure 2b). In contrast, the NPs group exhibited marked hepatic histopathological alterations. Inflammatory cell infiltration was observed exclusively in this group (Figure 2C). Compared with the control group, the NPs group showed nucleolar disappearance, mitochondrial swelling, marked mitochondrial cristae disorganization, and distinct cytoplasmic vacuolization (Figure 2c). In the NCUR + NPs group, the hepatic structure appeared improved compared with the NPs group. No obvious mitochondrial swelling or cytoplasmic vacuolization was detected (Figure 2d), and the overall structure was more similar to that of the control group (Figure 2D).

**Figure 3:**
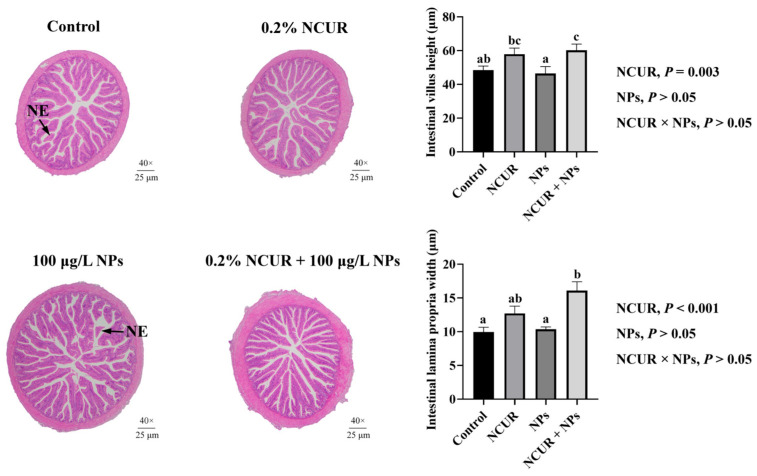
Histopathological changes in intestines observed in *M. salmoides* after exposure to NPs in water and fed with NCUR for 21 days. Necrosis and erosion (NE). Bar = 25 µm, magnification × 40. Different superscript letters (a, b, c) above the bars indicate significant differences among groups (*p* < 0.05).

In the intestines, compared with the NPs group, the NCUR + NPs group exhibited less apparent intestinal injury and a more intact structure. The intestinal villi heights of the NCUR group and the NCUR + NPs group were significantly higher than those in the NPs group (*p* < 0.05), while the intestinal lamina propria thickness of these two groups was significantly higher than that in the control group and the NPs group (*p* < 0.05). Two-way ANOVA indicated that the interactions between NPs and NCUR had no significant impact on intestinal villi height or lamina propria thickness (*p* > 0.05) (Figure 3).

### 3.3. Serum Biochemical Analysis

**Figure 4:**
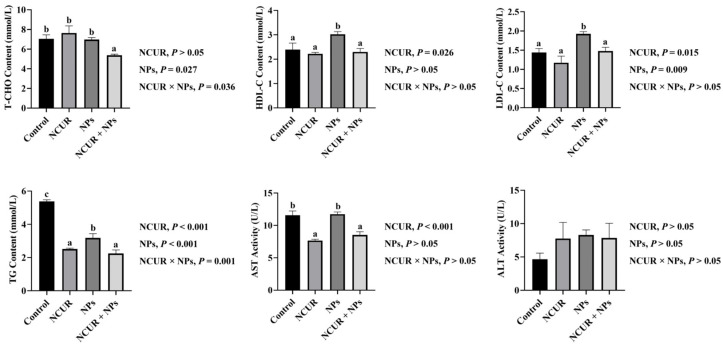
Effects of exposure to 100 μg/L NPs in water and dietary supplementation with 0.2% NCUR on the serum biochemical indices of *M. salmoides* after a 21-day trial (*n* = 3 tank replicates per treatment). Different superscript letters (a, b, c) above the bars indicate significant differences among groups (*p* < 0.05).

After a 21-day trial, the effects of 100 μg/L NPs and dietary supplementation with 0.2% NCUR on the serum biochemical indices of *M. salmoides* are presented in Figure 4.

For cholesterol metabolism, the serum HDL-C and LDL-C contents in the NPs group were significantly higher than those in the control, NCUR, and NCUR + NPs groups (*p* < 0.05), whereas the serum T-CHO content in the NCUR + NPs group was significantly lower than that in the control, NCUR, and NPs groups (*p* < 0.05).

For triglyceride metabolism, the serum TG content in the NPs group was significantly lower than that in the control group (*p* < 0.05). Additionally, the TG content in both the NCUR and NCUR + NPs groups was significantly decreased compared with the control and NPs groups (*p* < 0.05).

For hepatic function indicators, the AST activity in the serum of the NCUR and NCUR + NPs groups was significantly decreased compared with the control and NPs groups (*p* < 0.05). In contrast, ALT activity showed no significant difference among all groups (*p* > 0.05).

Two-way ANOVA revealed significant interactions between NPs and NCUR for serum T-CHO and TG contents (*p* = 0.036, *p* = 0.001, respectively).

### 3.4. Liver Antioxidant Capacity Analysis

**Figure 5:**
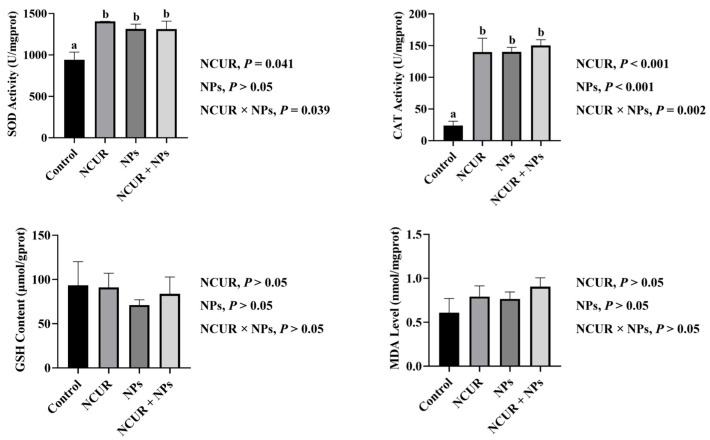
Effects of exposure to 100 μg/L NPs in water and dietary supplementation with 0.2% NCUR on hepatic oxidative stress in *M. salmoides* after a 21-day trial (*n* = 3 tank replicates per treatment). Different superscript letters (a, b) above the bars indicate significant differences among groups (*p* < 0.05).

Following the 21-day exposure trial, changes in the hepatic antioxidant capacity of *M. salmoides* in response to 100 μg/L waterborne NPs and 0.2% dietary NCUR supplementation are illustrated in Figure 5.

For the antioxidant defense enzymes, the hepatic SOD and CAT activities in the control group were significantly lower than those in the NCUR, NPs, and NCUR + NPs groups (*p* < 0.05), with no significant differences observed among the NCUR, NPs, and NCUR + NPs groups.

For the associated oxidative stress indicators, there were no statistically significant differences in hepatic GSH and MDA contents among all groups.

Two-way ANOVA revealed that the interaction between NPs and NCUR had a significant effect on hepatic SOD and CAT activities (*p* = 0.039, *p* = 0.002).

### 3.5. Expression of Liver-Related Genes

**Figure 6:**
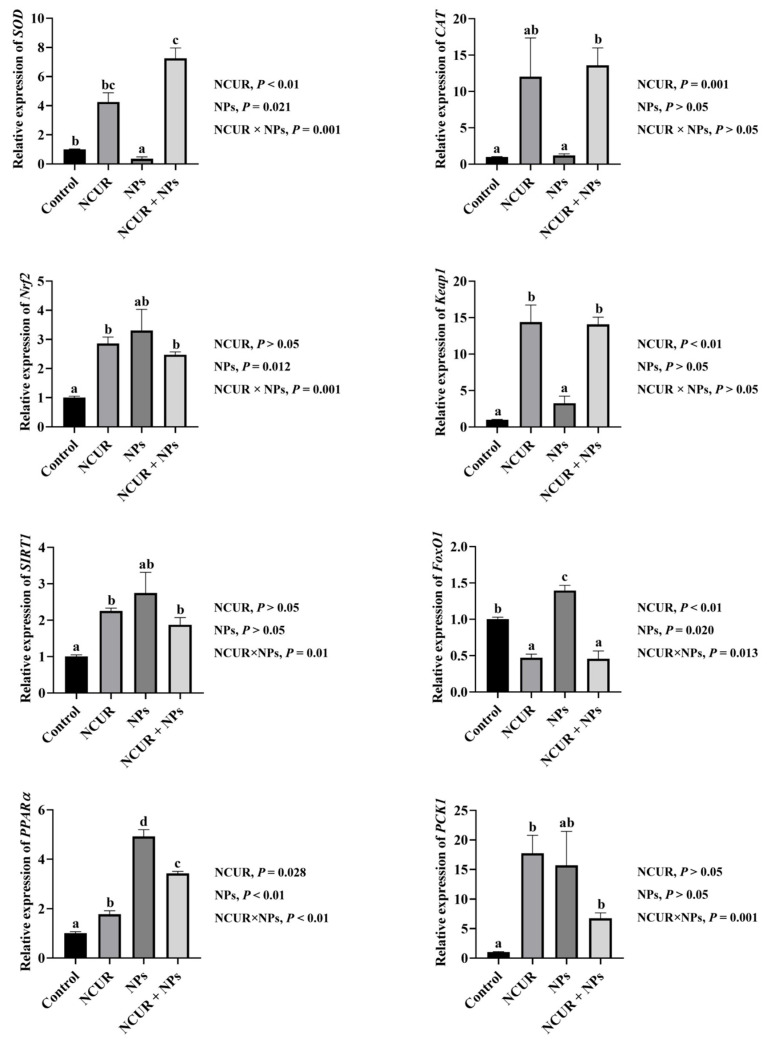
Effects of exposure to 100 μg/L NPs in water and dietary supplementation with 0.2% NCUR on the relative expression levels of hepatic genes in *M. salmoides* after a 21-day trial (*n* = 3 tank replicates per treatment). Different superscript letters (a, b, c, d) above the bars indicate significant differences among groups (*p* < 0.05).

As illustrated in Figure 6, 21 days of exposure to 100 μg/L waterborne NPs and dietary supplementation with 0.2% NCUR modulated the relative mRNA expression levels of hepatic genes in *M. salmoides*.

For genes involved in the antioxidant response, hepatic SOD expression was significantly lower in the NPs group than in the control group (*p* < 0.05). In contrast, hepatic SOD, CAT, and Keap1 expression levels were significantly higher in the NCUR + NPs group than in the NPs and control groups (*p* < 0.05).

For genes related to lipid metabolism and energy homeostasis, hepatic FoxO1 and PPARα expression levels were significantly higher in the NPs group than in the control, NCUR, and NCUR + NPs groups (*p* < 0.05). Meanwhile, hepatic Nrf2, Keap1, SIRT1, PPARα, and PCK1 expression levels were significantly upregulated in the NCUR and NCUR + NPs groups compared with the control group (*p* < 0.05).

Two-way ANOVA results showed significant interactions between NPs and NCUR for hepatic SOD, Nrf2, SIRT1, FoxO1, PPARα, and PCK1 expression levels (*p* < 0.05).

### 3.6. Intestinal Microbiota

#### 3.6.1. Richness and Diversity Analysis

After the 21-day trial, intestinal microbiota analysis was performed to describe microbial richness, diversity, and compositional patterns among treatments. Because two fish-level intestinal samples were collected from each tank, microbiota-related results were interpreted cautiously with consideration of the tank-based experimental design.

**Figure 7:**
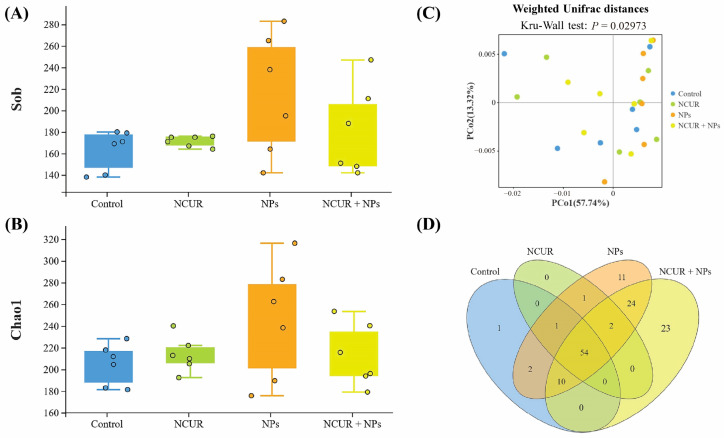
Effects of NCUR on the intestinal microbiota of *M. salmoides* exposed to 100 μg/L NPs in water. Boxplot of the Sobs index (**A**) and Chao1 index (**B**); Principal coordinate analysis (PCoA) based on Weighted UniFrac distances (**C**); Venn diagram showing the numbers of unique genera and shared genera among different groups (**D**). Each point represents one fish-level intestinal microbiota sample. Two fish-level samples were collected from each tank, and each treatment contained three replicate tanks. Fish sampled from the same tank were considered biological subsamples rather than independent tank-level replicates.

For alpha diversity indices (reflecting within-community richness and diversity), no significant differences were detected in the Sobs (Figure 7A) and Chao1 (Figure 7B) indices among all experimental groups (*p* > 0.05).

For beta diversity, weighted UniFrac-based PCoA showed visible differences in the distribution patterns of intestinal microbiota samples among treatments (*p* = 0.02973; Figure 7C).

Venn diagram analysis further characterized the compositional uniqueness of the microbiota: compared with the control group, the NCUR group, NPs group, and NCUR + NPs group harbored 0, 11, and 23 unique bacterial genera, respectively (Figure 7D).

#### 3.6.2. Composition and Functional Changes in Intestinal Microbiota

**Figure 8:**
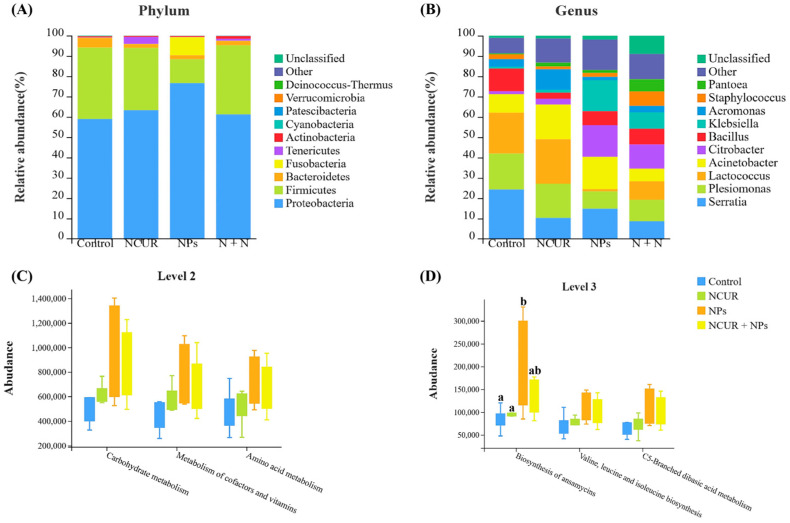
Effects of NCUR on composition and functional changes in intestinal microbiota of *M. salmoides* exposed to 100 μg/L NPs in water. Intestinal microbiota composition at the phylum level (**A**) and genus level (**B**); Functional prediction results at PICRUSt2 Level 2 (**C**) and Level 3 (**D**) of intestinal microbiota in different groups (different lowercase letters indicate differences among fish-level microbiota samples based on the applied statistical test, *p* < 0.05). Microbiota composition and predicted functional profiles were based on fish-level intestinal microbiota samples. Two fish-level samples were collected from each tank, and results were interpreted cautiously with consideration of the tank-based experimental design.

At the phylum level, Proteobacteria, Firmicutes, and Actinobacteriota were the dominant phyla in the control group, accounting for 58.95%, 35.17%, and 4.74%, respectively. NP exposure alone was associated with an increased relative abundance of Proteobacteria (76.60%) and decreased Firmicutes (11.76%), with Fusobacteria (8.86%) also observed as a major phylum. In contrast, the NCUR + NPs group showed a compositional pattern more similar to the control group than to the NPs group, with Proteobacteria (61.21%), Firmicutes (34.05%), and Actinobacteriota (2.10%) as the dominant phyla (Figure 8A).

At the genus level, NP exposure alone increased the relative abundances of *Acinetobacter*, *Citrobacter*, *Klebsiella*, and other genera, while decreasing those of *Serratia*, *Plesiomonas*, *Bacillus*, and *Lactococcus*. NCUR supplementation alone elevated the relative abundances of *Acinetobacter* and *Aeromonas*, and reduced those of *Serratia* and *Bacillus*. When combined with NP exposure, NCUR supplementation resulted in increased relative abundances of *Citrobacter*, *Klebsiella*, *Pantoea*, and *Staphylococcus*, along with reduced abundances of *Serratia*, *Plesiomonas*, *Lactococcus*, and *Bacillus* (Figure 8B).

The potential functional prediction of the intestinal microbiota was analyzed using PICRUSt2 software, and the results showed that the ansamycin biosynthesis pathway was significantly enriched in the NPs group compared with the control group and NCUR group (Figure 8D). Because PICRUSt2 provides predicted rather than directly measured functions, and because microbiota samples were fish-level subsamples within tanks, this result should be interpreted cautiously.

## 4. Discussion

In this study, after a 21-day aquaculture trial, the FBW, WGR and SGR of fish in the NPs group were significantly lower than those in the control group. Meanwhile, the FI in the NPs group was significantly decreased compared with that in the control group. This phenomenon may be related to reduced feeding activity or impaired digestive function following NP exposure, as nanoplastic uptake and accumulation can affect intestinal function and feeding behavior in fish [25]. In the present study, no significant difference was observed in growth parameters between the NCUR + NPs group and the control group, and the NCUR + NPs group showed intermediate values for FBW, WGR, and SGR compared with the control and NPs groups. These results suggest that dietary NCUR was associated with a partial improvement in growth-related parameters under NP exposure. Nevertheless, it should be interpreted cautiously, because the NPs × NCUR interaction was not significant for growth performance. In addition, the NCUR-only group showed changes in some biological endpoints, suggesting that the independent effects of NCUR should also be considered when evaluating its role as a feed additive. Although few studies have focused on NCUR as a dietary additive for alleviating pollutant-induced toxic effects in aquatic animals, our findings are broadly consistent with previous research on CUR mitigating pollutant-associated growth impairment in aquatic animals. García-Pérez et al. [18] found that dietary supplementation with 200 mg/kg CUR in aflatoxin-contaminated feed significantly increased the FI and growth rate in *Penaeus vannamei*. Giri et al. [19] reported that dietary CUR supplementation improved growth performance and feed utilization-related parameters in *C. carpio* exposed to Pb. In the present study, NP-associated growth impairment in *M. salmoides* may be partly linked to histopathological alterations observed in key tissues, including the gills, liver, and intestine.

In terms of tissue pathological changes, gill lamellar curvature and epithelial and basal cell hyperplasia were observed in both the NPs group and the NCUR + NPs group, indicating that NCUR did not visibly alleviate NPs-induced gill tissue injury in *M. salmoides* after 21 days of feeding. Importantly, the NCUR-only group also exhibited branchial histopathological alterations, including lamellar fusion, basal cell hyperplasia and aneurysm. This finding suggests that NCUR may exert tissue-specific or carrier-related effects under the present experimental conditions and should not be interpreted solely as a protective additive. Because the gills are in direct contact with the aquatic environment and are highly sensitive to suspended particles, dietary additives, dissolved compounds, and liposomal carriers, the branchial response observed in the NCUR-only group deserves further attention. At present, the mechanism underlying this response remains unclear, and further quantitative histopathological scoring, dose–response assessment, and carrier-control experiments are needed to evaluate the safety of NCUR supplementation. The hepatic and intestinal histological results showed that NP exposure was associated with hepatic inflammatory cell infiltration, lipid droplet accumulation, hepatic nucleolus disappearance, mitochondrial swelling, mitochondrial cristae disorder and cytoplasmic vacuolization, as well as intestinal villus necrosis and erosion in *M. salmoides*. Compared with the NPs group, the NCUR + NPs group showed less apparent hepatic and intestinal structural disruption, suggesting that NCUR may help maintain hepatic and intestinal tissue integrity under NP exposure. Consistent with the current findings, Atia et al. [26] reported that both NCUR and CUR could alleviate acrylamide-induced hepatic injury in mice, while NCUR enhanced the hepatoprotective effect of CUR. The findings of Tohamy et al. [27] showed that NCUR could alleviate nanocopper-induced liver injury in mice. Wang et al. [28] reported that CUR and composite polysaccharide microparticles partially restored intestinal barrier integrity in DSS-induced ulcerative colitis mice by alleviating ZO-1 disruption, with the combined treatment showing the strongest effect. Similarly, Mohamed et al. [29] reported that although mild hepatic degeneration and inflammation were still observed in the CUR + Cr^6+^ group, CUR reduced the frequency and severity of Cr^6+^-induced hepatic lesions in Nile tilapia. Together, these observations suggest potential tissue-protective effects of NCUR under pollutant exposure, but the branchial changes observed in the NCUR-only group indicate that its safety profile requires further investigation.

Serum biochemical indices are important biomarkers for evaluating hepatic lipid metabolism and functional damage. In the present study, the levels of T-CHO, HDL-C, LDL-C, and TG in the NCUR + NPs group were significantly lower than those in the NPs group, suggesting that NCUR supplementation may influence serum lipid-related parameters under NP exposure. Meanwhile, the AST activity in the NCUR group and NCUR + NPs group was significantly decreased compared with that in the NPs group and control group, whereas ALT activity did not differ significantly among groups. Therefore, the serum biochemical results suggest a partial ameliorative effect of NCUR on NP-associated metabolic disturbances, rather than comprehensive evidence of complete hepatic protection. Two-way ANOVA further revealed a significant interaction between NPs and NCUR on serum T-CHO and TG contents, indicating that the effect of NCUR on serum lipid-related parameters depended, at least in part, on NP exposure. These effects may be associated with concurrent changes in hepatic antioxidant enzyme activities and metabolism-related gene expression.

CUR is a plant-derived natural compound with antioxidant-related bioactivities and may contribute to the regulation of endogenous antioxidant responses, as reflected by changes in hepatic antioxidant enzyme activities and redox-related gene expression. In this study, hepatic SOD and CAT activities were significantly higher in the NCUR, NPs, and NCUR + NPs groups than in the control group, whereas hepatic GSH and MDA contents did not differ significantly among groups. These findings suggest that, under the present experimental conditions, NP exposure and NCUR supplementation mainly elicited an adaptive antioxidant enzyme response rather than providing clear evidence of severe oxidative damage or lipid peroxidation. Partly consistent with the present results, Abdel-Tawwab et al. [30] reported that dietary curcumin nanoparticles enhanced antioxidant enzyme activities, including SOD, CAT, and GPx, in Nile tilapia fingerlings. The findings of Yonar [31] revealed that the chlorpyrifos + CUR group significantly enhanced the hepatic SOD and GST activities of *C. carpio* compared with the control group. Zhang et al. [32] reported that the hepatic SOD activity in *C. carpio* of the CCl_4_ group and CCl_4_ + CUR group was significantly higher than that in the control group, whereas no significant differences were observed in GSH content among all groups. Nrf2 is a key transcriptional regulator of antioxidant genes and is controlled by Keap1, a negative regulator that promotes Nrf2 ubiquitination and proteasomal degradation under basal conditions. In response to oxidative or electrophilic stimuli, Nrf2 may dissociate from Keap1 and translocate to the nucleus, where it regulates antioxidant response element (ARE)-dependent genes [33]. In the present study, the mRNA expression levels of Nrf2 and Keap1 were significantly higher in the NCUR and NCUR + NPs groups than in the control group. In addition, the mRNA expression levels of SOD, CAT, and Keap1 were markedly increased in the NCUR + NPs group compared with the NPs and control groups. These transcriptional changes suggest that Nrf2/Keap1-related antioxidant responses may be involved in the effects of NCUR. However, because Keap1 is generally considered a negative regulator of Nrf2, increased Keap1 mRNA expression should not be interpreted as direct evidence of Nrf2 pathway activation, but may reflect feedback or compensatory regulation. Moreover, Nrf2 protein abundance, nuclear translocation, and downstream protein targets such as HO-1 and NQO1 were not assessed in the present study. Therefore, the current data support transcriptional modulation of Nrf2/Keap1-associated genes, but do not directly demonstrate activation of the Nrf2/Keap1 pathway. Partly consistent with the findings of the present study, Kheiripour et al. [34] found that CUR and NCUR alleviated paraquat-induced liver injury in mice through mechanisms involving Nrf2 signaling, and NCUR exerted a better hepatoprotective effect than CUR. In fish, Zhang et al. [35] reported that dietary CUR enhanced antioxidant-related responses and alleviated CCl_4_-induced hepatic damage in *C. carpio*, possibly through the Nrf2 signaling pathway.

SIRT1, a multifunctional protein deacetylase that integrates antioxidant stress and metabolic regulation, has attracted considerable attention due to its roles in cellular stress responses; the involved mechanisms include the SIRT1/FoxOs, SIRT1/NF-κB, SIRT1/NOX, SIRT1/SOD, and SIRT1/eNOS pathways [36]. SIRT1 can interact with the downstream signaling protein FoxO1 and participate in FoxO1 deacetylation, thereby affecting the transcription of genes involved in apoptosis, antioxidant defense and metabolism [37]. PPARα is the major hepatic transcriptional regulator of organismal metabolism, and participates in energy utilization and the transcription of metabolism-related factors (e.g., FGF21, FADS, PCK1) [38]. In the present study, the expression levels of FoxO1 and PPARα genes in the NPs group were markedly higher than those in the control, NCUR, and NCUR + NPs groups. In contrast, SOD gene expression in the NPs group was significantly lower than that in the control, NCUR, and NCUR + NPs groups. These results, together with the alterations in lipid-related serum biochemical parameters, suggest that NP exposure was associated with hepatic redox and lipid metabolic disturbances in *M. salmoides*. Consistent with the findings of the present study, Brandts et al. [39] reported that exposure to 45 nm polymethyl methacrylate (PMMA) nanoplastics in water for 96 h increased the hepatic PPARα and PPARγ gene expression levels in *Sparus aurata* but had no significant effect on PPARβ gene expression. In the present study, the expression levels of SIRT1, PPARα, and PCK1 genes in the NCUR group and NCUR + NPs group were significantly higher than those in the control group, whereas FoxO1 gene expression in the NCUR group and NCUR + NPs group was markedly decreased compared with that in the control and NPs group. Collectively, these results indicate that NCUR may modulate the transcription of SIRT1/FoxO1- and PPARα-related genes, which may be associated with changes in hepatic antioxidant enzyme activities and lipid metabolism under NP exposure. However, because only mRNA expression was measured, these findings should be interpreted as transcriptional associations rather than direct evidence of SIRT1/FoxO1 pathway activation. Protein-level validation, analysis of FoxO1 acetylation or phosphorylation, and pathway-specific functional experiments are needed to confirm this mechanism. Consistent with this cautious interpretation, Cui et al. [40] reported that curcumin regulated SIRT1- and FoxO1-related responses in porcine renal epithelial cells under zearalenone-induced oxidative injury. Hou et al. [41] further found that curcumin promotes mitochondrial biogenesis and inhibits mitochondrial fragmentation by activating SIRT1 in a mouse sepsis model. These studies support the potential involvement of SIRT1-related stress responses in the antioxidant effects of curcumin, although direct evidence for SIRT1/FoxO1 pathway activation was not obtained in the present study.

The intestinal microbiota is an important component of fish microecological balance and is closely associated with tissue integrity and metabolic status. In the present study, intestinal microbiota analysis suggested treatment-associated changes in microbial compositional patterns. The NPs group showed changes in the dominant phyla of the intestinal microbiota in *M. salmoides*, whereas the dominant phyla in the NCUR + NPs group showed a compositional pattern more similar to that of the control group. Functional prediction using PICRUSt2 showed that the ansamycin biosynthesis pathway was enriched in the NPs group compared with the control and NCUR groups, whereas no significant difference was observed between the NCUR + NPs group and the control group. This predicted enrichment may reflect changes in microbial functional potential related to secondary metabolite production. However, because PICRUSt2 provides inferred rather than directly measured functions, these results require further validation by metagenomic, metabolomic, or targeted functional analyses. Overall, these findings suggest that NP exposure was associated with changes in intestinal microbiota composition and predicted microbial functional potential, while NCUR supplementation may partially modulate these microbiota-related patterns under NP exposure. It should also be noted that the NCUR-only group showed changes in the relative abundance of several genera, including increased *Acinetobacter* and *Aeromonas* and decreased *Serratia* and *Bacillus*. Therefore, the intestinal microbiota results should not be interpreted solely as restoration of microbial homeostasis, but rather as evidence that NCUR itself may influence intestinal microbial composition under the present experimental conditions. Khorshidi et al. [42] reported that treatment with 750 mg/kg CUR for 60 days mitigated silver nanoparticle-induced toxicity in *C. carpio*, particularly the reduction in the total number of intestinal microbes. This finding suggests that curcumin-based additives may influence gut microbiota under nanoparticle exposure; however, the biological significance of the specific microbial shifts observed in the present study requires further validation.

Several limitations should be acknowledged. First, the proposed involvement of Nrf2/Keap1 and SIRT1/FoxO1 signaling was inferred mainly from mRNA expression changes and antioxidant enzyme activities. Protein-level validation, Nrf2 nuclear translocation, phosphorylation or acetylation analyses, and pathway-specific functional assays were not performed; therefore, pathway activation cannot be directly concluded. Second, although SOD and CAT activities were altered, hepatic GSH and MDA contents did not differ significantly among groups, suggesting that the present exposure conditions may have induced an adaptive redox response rather than overt oxidative injury. Third, the branchial lesions observed in the NCUR-only group indicate that the independent effects of NCUR, its optimal dosage, potential liposomal carrier effects, and long-term safety require further evaluation. Finally, the use of commercially available pristine PS-NPs provided a controlled exposure model, but may not fully represent environmentally aged nanoplastics encountered in aquaculture systems. In addition, direct quantitative monitoring of background nanoplastic contamination in the experimental water was not performed. For intestinal microbiota analysis, fish-level samples collected from the same tank were considered biological subsamples, and therefore microbiota-related findings should be further validated using tank-level replication and complementary metagenomic or metabolomic approaches.

## 5. Conclusions

This study investigated the effects of 100 μg/L waterborne NPs on *M. salmoides* and the potential of dietary 0.2% NCUR to modulate NP-associated biological disturbances. NP exposure inhibited growth and feed intake, induced gill, hepatic and intestinal pathological alterations, and was accompanied by serum lipid changes, altered antioxidant enzyme activities, transcriptional changes in SOD, FoxO1 and PPARα, and apparent changes in intestinal microbiota compositional patterns. Under NP exposure, dietary NCUR partially modulated selected NP-associated responses, including hepatic and intestinal structural alterations, serum lipid disturbances, and changes in selected antioxidant- and metabolism-related gene transcripts. However, the present evidence supports transcriptional modulation of Nrf2/Keap1- and SIRT1/FoxO1-related genes rather than direct pathway activation. Notably, NCUR did not alleviate NPs-induced gill damage, and NCUR alone induced branchial histopathological alterations, indicating that its independent effects and safety profile require further investigation. Overall, NCUR may have potential as a dietary additive to partially mitigate selected NP-associated disturbances in *M. salmoides*, but dose optimization, carrier-control evaluation, protein-level validation and longer-term safety studies are needed before practical application.

## Figures and Tables

**Table 1:** Formulation and nutritional composition of experimental diets (%, dry matter).

Ingredients	Basal Diet	NCUR Diet
Fish meal	40	40
Soybean meal	10.5	10.5
Peanut meal	9	9
Wheat flour	10	10
Vital wheat gluten	9.5	9.5
Beer yeast	3	3
Fish oil	3	3
Soya lecithin	1	1
Choline chloride	0.5	0.5
Monocalcium phosphate	1.5	1.5
Gunk ^1^	2	2
Avicel	10	9
Nanocurcumin (20%) ^2^	0	1
Proximate composition ^3^		
Moisture	7.86	6.80
Crude protein	30.07	30.33
Crude lipid	11.17	12.26
Ash	18.88	18.79

Notes: ^1^ Gunk: The vitamin mixture (IU or mg/kg diet) consisted of VA 250,000 IU, VD3 45,000 IU, VC 7000 mg, riboflavin 750 mg, cyanocobalamin 1 mg, thiamine 250 mg, pyridoxine hydrochloride 400 mg, menadione 250 mg, folic acid 125 mg, biotin 10 mg, α-tocopherol 2.5 g, inositol 8000 mg, calcium pantothenate 1250 mg, niacin 2000 mg, and choline chloride 8000 mg. The mineral mixture (mg/kg diet) included NaCl 2.6 g, KCl 5.3 g, CaCO_3_ 37.9 g, KI 0.04 g, CuSO_4_·5H_2_O 0.02 g, ZnSO_4_·7H_2_O 0.04 g, CoSO_4_·7H_2_O 0.02 g, FeSO_4_·7H_2_O 0.9 g, MnSO_4_·H_2_O 0.03 g, Ca(HPO_4_)_2_·2H_2_O 9.8 g, and MgSO_4_·7H_2_O 3.5 g. Both mixtures were provided by Guangzhou Chengyi Aquatic Co., Ltd. (Guangzhou, China). ^2^ Nanocurcumin: The carrier was a liposome with a particle size of 70 nm, an encapsulation efficiency greater than 95%, and a drug-loading capacity of 20%, purchased from Shanghai Yihu Biotechnology Co., Ltd. (Shanghai, China). ^3^ Approximate composition values are based on actual measurements.

**Table 2:** Primer sequences.

Gene	Forward Primer (5′-3′)	Reverse Primer (5′-3′)
*β-actin*	AAAGGGAAATCGTGCGTGAC	AAGGAAGGCTGGAAGAGGG
*CAT*	GTTCCCGTCCTTCATCCACT	CAGGCTCCAGAAGTCCCACA
*FoxO1*	AGGAGACCGAGGACTTTACG	TGATGATGCGGGAGTTGC
*Keap1*	CAGCATTACATGGCCGCATC	CTTCTCTGGGTCGTAAGACTCC
*Nrf2*	CAGACAGTTCCTTTGCAGGC	AGGGACAAAAGCTCCATCCA
*PCK1*	TGTAACCCCGAGCAGACATT	CCAGATTTGTCTTCCCGCAG
*PPARα*	CCACCGCAATGGTCGATATG	TGCTGTTGATGGACTGGGAAA
*SIRT1*	TGGATTGTGAGGCTGTAAGG	ATGAGGAATGGAGTTTGGGA
*SOD*	TGGCAAGAACAAGAACCACA	CCTCTGATTTCTCCTGTCACC

Notes: *CAT*: catalase; *FoxO1*: forkhead box o1; *Keap1*: kelch-like ECH-associated protein 1; *Nrf2*: nuclear factor erythroid 2-related factor 2; *PCK1*: phosphoenolpyruvate carboxykinase 1; *PPARα*: peroxisome proliferator-activated receptor α; *SIRT1*: sirtuin 1; *SOD*: superoxide dismutase.

**Table 3:** Growth performance of *M. salmoides* exposed to 100 μg/L NPs in water and fed with 0.2% NCUR for 21 days.

Time	Items	Control	NCUR	NPs	NCUR + NPs
7-day	IBW (g)	11.53 ± 0.01	11.51 ± 0.01	11.52 ± 0.01	11.53 ± 0.01
	FBW (g)	12.83 ± 0.17 ^ab^	13.13 ± 0.27 ^b^	12.43 ± 0.21 ^ab^	12.12 ± 0.19 ^a^
	WGR (%)	11.27 ± 1.32 ^ab^	14.03 ± 2.48 ^b^	7.84 ± 1.79 ^ab^	5.10 ± 1.68 ^a^
	SGR (%/day)	1.52 ± 0.17 ^ab^	1.87 ± 0.31 ^b^	1.07 ± 0.24 ^ab^	0.71 ± 0.23 ^a^
	HSI (%)	2.77 ± 0.15	2.86 ± 0.09	2.43 ± 0.48	2.77 ± 0.17
	VSI (%)	7.53 ± 0.32	7.07 ± 0.45	6.52 ± 0.67	6.46 ± 0.35
	CF (g/cm^3^)	1.49 ± 0.03	1.45 ± 0.04	1.51 ± 0.05	1.46 ± 0.05
	FI per fish (g)	1.93 ± 0.05	1.91 ± 0.12	1.77 ± 0.07	1.69 ± 0.09
14-day	FBW (g)	13.78 ± 0.25	13.76 ± 0.21	13.90 ± 0.15	13.32 ± 0.03
	WGR (%)	19.51 ± 2.06	19.46 ± 1.89	20.66 ± 1.23	15.52 ± 0.31
	SGR (%/day)	1.27 ± 0.12	1.27 ± 0.11	1.34 ± 0.07	1.03 ± 0.02
	HSI (%)	2.72 ± 0.66	3.49 ± 0.58	3.46 ± 0.54	3.81 ± 0.22
	VSI (%)	7.90 ± 0.52	7.09 ± 1.03	7.24 ± 0.64	7.43 ± 0.55
	CF (g/cm^3^)	1.43 ± 0.03	1.48 ± 0.06	1.44 ± 0.03	1.48 ± 0.04
	FI per fish (g)	4.58 ± 0.14	4.72 ± 0.34	4.23 ± 0.18	4.12 ± 0.14
21-day	FBW (g)	15.91 ± 0.22 ^b^	15.95 ± 0.40 ^b^	14.92 ± 0.22 ^a^	15.36 ± 0.13 ^ab^
	WGR (%)	38.01 ± 1.71 ^b^	38.52 ± 3.37 ^b^	29.50 ± 1.88 ^a^	33.21 ± 1.03 ^ab^
	SGR (%/day)	1.53 ± 0.06 ^b^	1.55 ± 0.12 ^b^	1.23 ± 0.07 ^a^	1.37 ± 0.04 ^ab^
	HSI (%)	3.69 ± 0.31	3.86 ± 0.66	3.49 ± 0.69	5.08 ± 0.85
	VSI (%)	7.76 ± 0.26	8.30 ± 0.51	7.64 ± 0.77	9.19 ± 1.16
	CF (g/cm^3^)	1.87 ± 0.05	1.91 ± 0.06	1.91 ± 0.03	2.02 ± 0.06
	FI per fish (g)	9.07 ± 0.31 ^b^	8.50 ± 0.62 ^ab^	7.75 ± 0.17 ^a^	7.86 ± 0.20 ^ab^
	FCR	2.07 ± 0.03	1.92 ± 0.05	2.30 ± 0.15	2.06 ± 0.08
	SR (%)	100.00 ± 0.00	100.00 ± 0.00	98.33 ± 0.83	98.33 ± 1.67

Notes: Data are expressed as mean ± SEM (*n* = 3 tank replicates per treatment). Different superscript letters within the same row indicate significant differences among groups based on Tukey’s HSD test (*p* < 0.05).

**Table 4:** Two-way ANOVA *p*-values for the effects of NCUR, NPs, and their interaction on growth performance of *M. salmoides*.

Time	Items	NCUR	NPs	NCUR × NPs
7-day	IBW (g)	/	/	/
	FBW (g)	ns	0.011	ns
	WGR (%)	ns	0.011	ns
	SGR (%/day)	ns	0.010	ns
	HSI (%)	ns	ns	ns
	VSI (%)	ns	ns	ns
	CF (g/cm^3^)	ns	ns	ns
	FI per fish (g)	ns	ns	ns
14-day	FBW (g)	ns	ns	ns
	WGR (%)	ns	ns	ns
	SGR (%/day)	ns	ns	ns
	HSI (%)	ns	ns	ns
	VSI (%)	ns	ns	ns
	CF (g/cm^3^)	ns	ns	ns
	FI per fish (g)	ns	ns	ns
21-day	FBW (g)	ns	0.016	ns
	WGR (%)	ns	0.013	ns
	SGR (%/day)	ns	0.013	ns
	HSI (%)	ns	ns	ns
	VSI (%)	ns	ns	ns
	CF (g/cm^3^)	ns	ns	ns
	FI per fish (g)	ns	0.030	ns
	FCR	ns	ns	ns
	SR (%)	ns	ns	ns

Notes: ns indicates no significant difference (*p* > 0.05). The *p*-values were obtained from two-way ANOVA evaluating the main effects of NCUR, NPs, and their interaction.

## Data Availability

All data generated or analyzed during this study are included in this published article. Primer sequences used for qPCR analysis are listed in Table 2 of the manuscript.

## Abbreviations

The following abbreviations are used in this manuscript:

English abbreviation	Definition
NPs	Nanoplastics
PS-NPs	Polystyrene nanoplastics
CUR	Curcumin
NCUR	Nanocurcumin
*M. salmoides*	*Micropterus salmoides*
WGR	Weight gain rate
SGR	Specific growth rate
FCR	Feed conversion ratio
HSI	Hepatosomatic index
VSI	Viscerosomatic index
CF	Condition factor
SR	Survival rate
SOD	Superoxide dismutase
CAT	Catalase
GSH	Reduced glutathione
MDA	Malondialdehyde
GSH-Px	Glutathione peroxidase
AST	Aspartate aminotransferase
ALT	Alanine aminotransferase
TCHO	Total cholesterol
TG	Triglyceride
HDL-C	High-density lipoprotein cholesterol
LDL-C	Low-density lipoprotein cholesterol
*Nrf2*	Nuclear factor erythroid 2-related factor 2
*Keap1*	Kelch-like ECH-associated protein 1
*SIRT1*	Sirtuin 1
*FoxO1*	Forkhead box O1
*PPARα*	Peroxisome proliferator-activated receptor α
*PCK1*	Phosphoenolpyruvate carboxykinase 1
ARE	Antioxidant response element
